# Supplementary figures and images for: Human IL-2Rɑ subunit binding modulation of IL-2 through a decline in electrostatic interactions: A computational and experimental approach
Source: PLoS One. 2022 Feb 25;17(2):e0264353. doi: 10.1371/journal.pone.0264353 (PMC8880607; doi:10.1371/journal.pone.0264353)

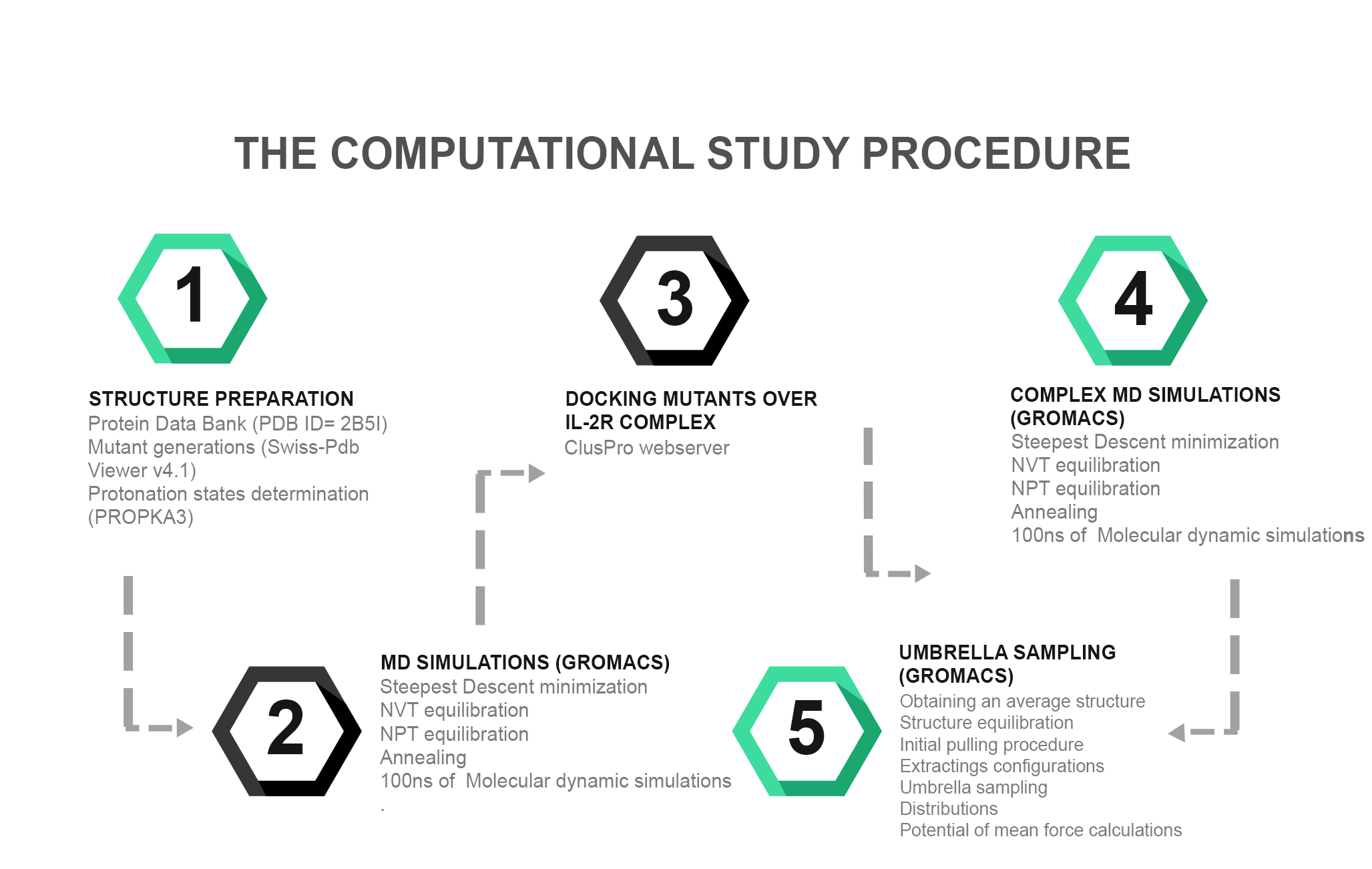

Supplement: S1 Fig — (TIF) [file pone.0264353.s001.tif]

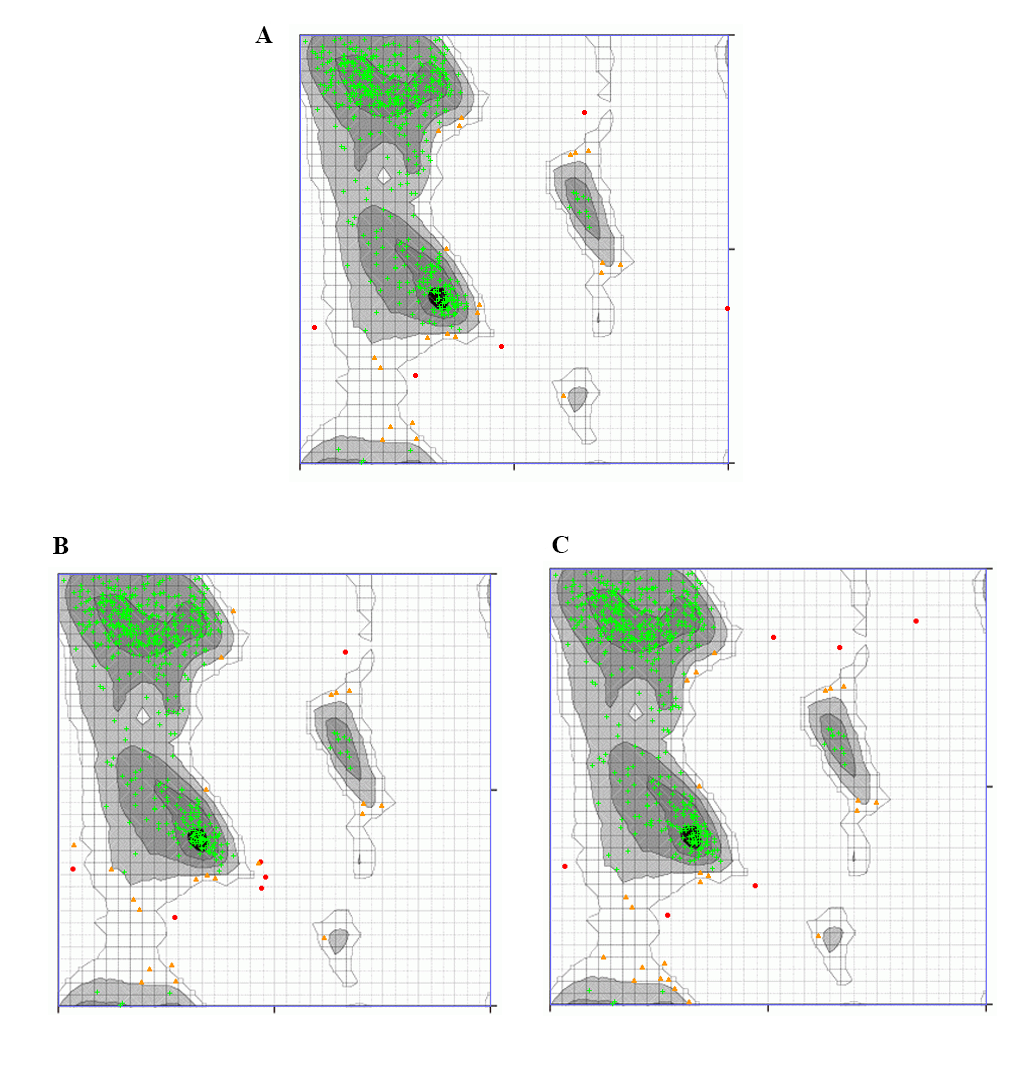

Supplement: S2 Fig — Ramachandran plots of the final A. wtIL-2, B. Mutant 1, and C. Mutant 2, after 100 ns of MDs. Highly preferred, preferred and questionable observations are shown as green crosses, brown triangles, and red circles, respectively. (TIF) [file pone.0264353.s002.tif]

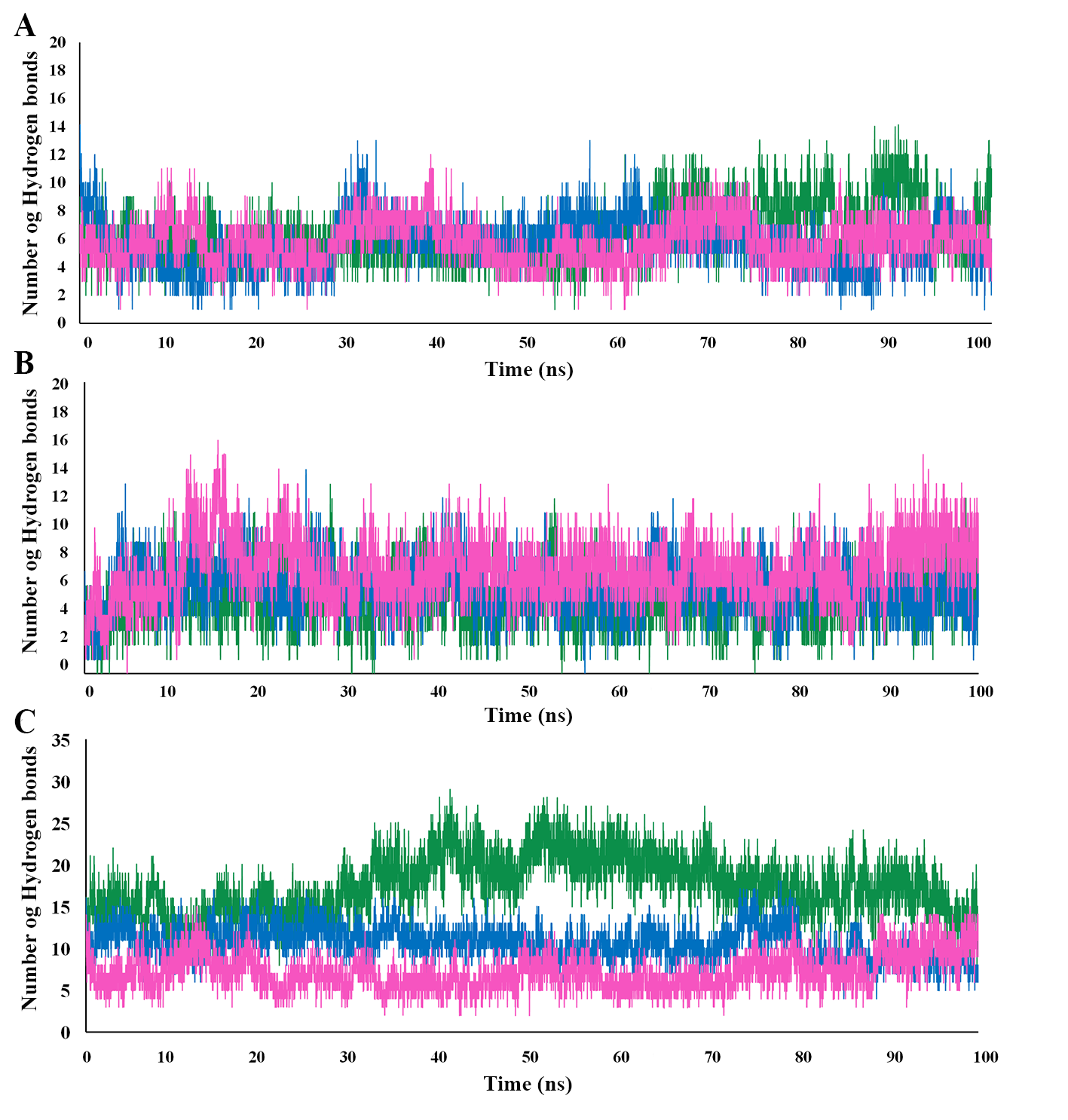

Supplement: S3 Fig — The number of hydrogen bonds between wtIL-2 (green), M1 (blue) and M2 (magenta) with A. IL-2Rβ, B. IL-2Rγc, and C. IL-2Rα. (TIF) [file pone.0264353.s003.tif]

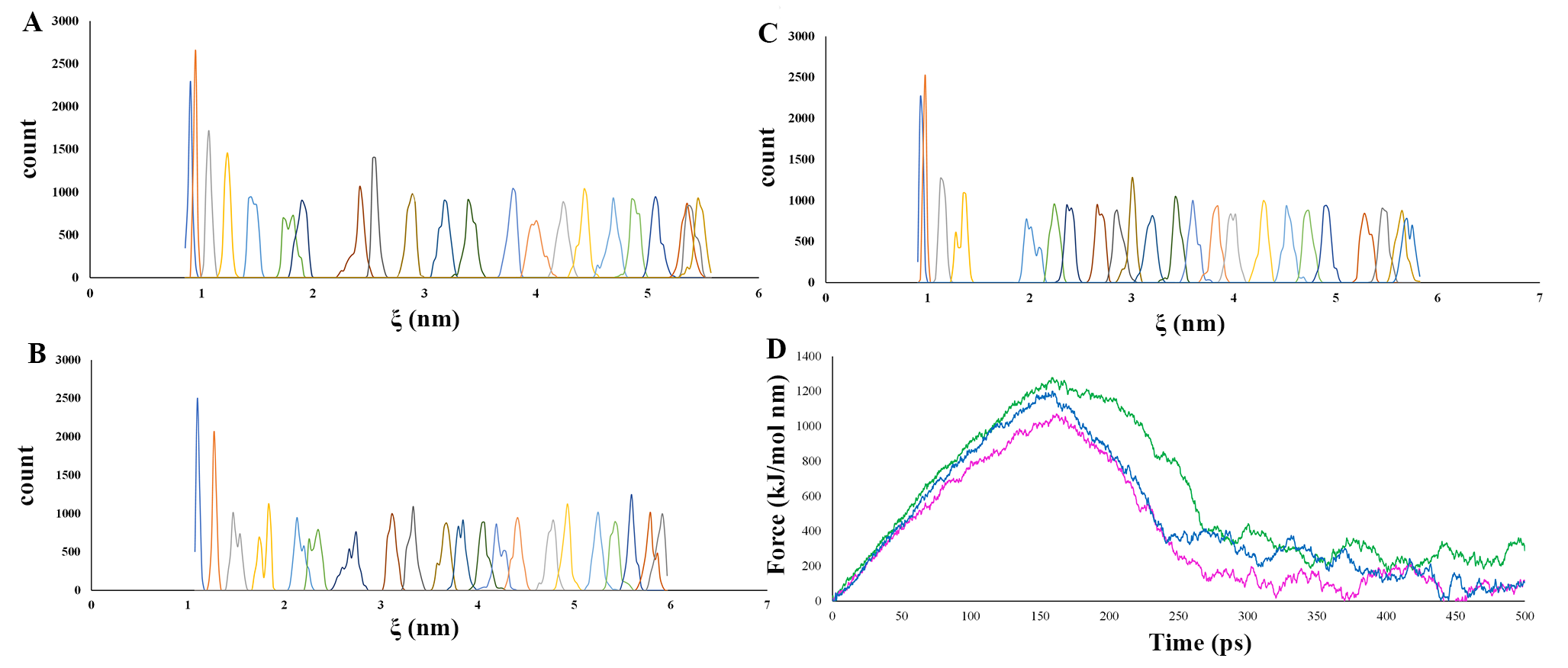

Supplement: S4 Fig — All umbrella sampling window simulations for, A. IL-2Rα begins dissociation from wtIL-2 (green), B. IL-2Rα begins dissociation from M1 (blue) and C. IL-2Rα begins dissociation from M2 (magenta). D. The force on the spring over 500 ps of MDs for IL-2Rα begins dissociation from wtIL-2 (green), M1 (blue) and M2 (magenta). (TIF) [file pone.0264353.s004.tif]

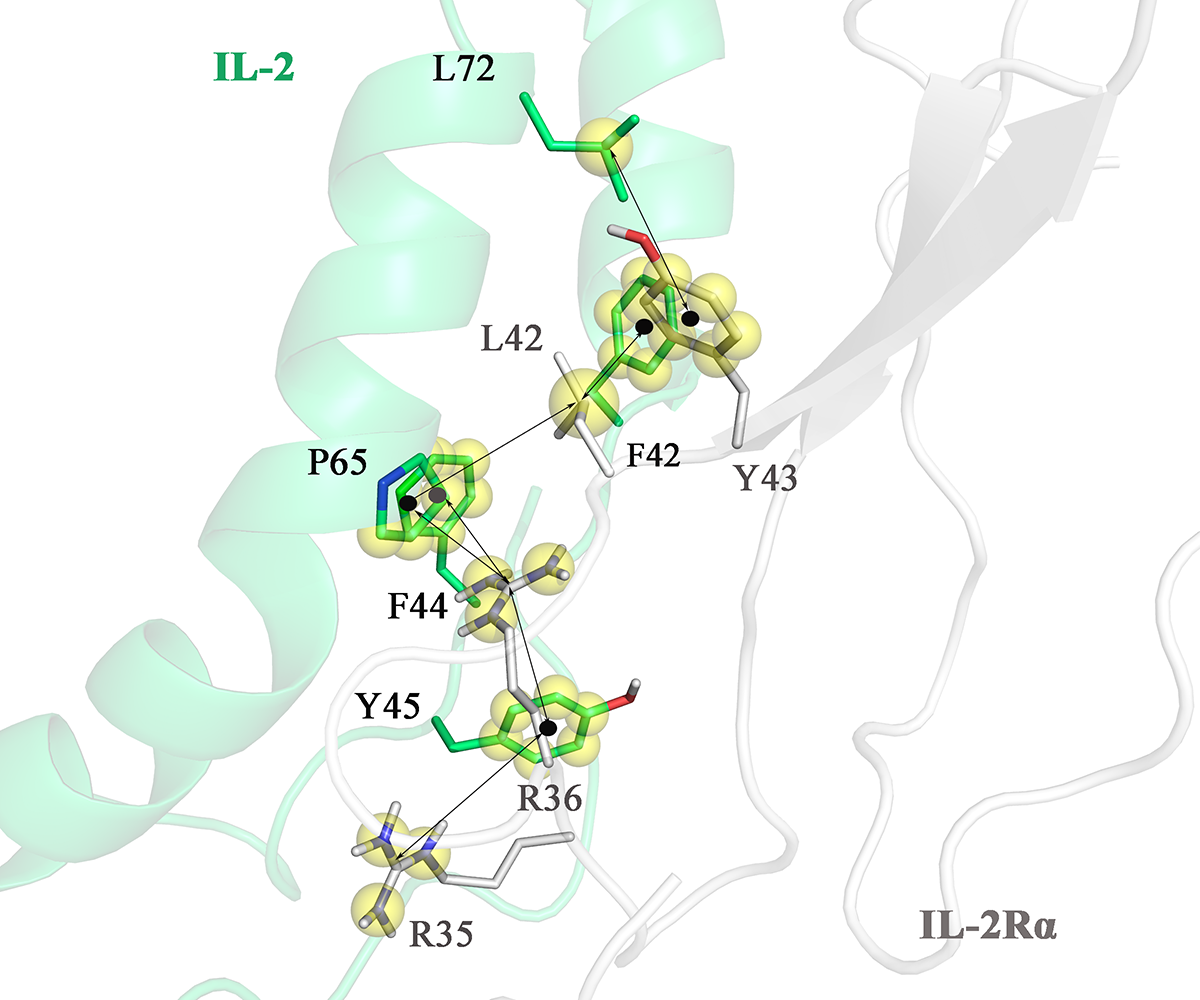

Supplement: S5 Fig — The close view of the A-B loop and the grooves between IL-2Rα strands, the hydrophobic ridges around residues F42, Y45, and L72 and their electrostatic interactions. The yellow shperes represent hydrophobic areas and the two end arrows represent electrostatic (like π-π, alkyl-π and π-cation) interactions. (TIF) [file pone.0264353.s005.tif]
