# Supplementary material for: Human IL-2Rɑ subunit binding modulation of IL-2 through a decline in electrostatic interactions: A computational and experimental approach
Source: PLoS One. 2022 Feb 25;17(2):e0264353. doi: 10.1371/journal.pone.0264353 (PMC8880607; doi:10.1371/journal.pone.0264353)
Supplement: S1 Table — (DOCX) [file pone.0264353.s006.docx]

| Point Mutation | ProDIGGY | | PISA |
| --- | --- | --- | --- |
|  | **ΔG (Kcal/mol)** | **Kd (M)** | **ΔG (Kcal/mol)** |
| wtIL-2 | -9.4 | 1.20E-07 | 1 |
| K35A | -7.5 | 3.20E-06 | 0.3 |
| R38A | -8.6 | 5.30E-07 | -0.9 |
| F42A | -8.6 | 5.30E-07 | 2 |
| K43A | -9 | 2.70E-07 | 0.4 |
| Y45A | -8.8 | 3.80E-07 | 0.3 |
| E61A | -8.3 | 7.90E-07 | 0.7 |
| E62A | -9.5 | 1.00E-07 | -1.2 |
| P65A | -9.7 | 7.7E-08 | 1.1 |
| L72A | -8.9 | 3.2E-07 | 1.6 |
| Y107A | -9.2 | 1.80E-07 | 0.7 |
