# Supplementary material for: Human IL-2Rɑ subunit binding modulation of IL-2 through a decline in electrostatic interactions: A computational and experimental approach
Source: PLoS One. 2022 Feb 25;17(2):e0264353. doi: 10.1371/journal.pone.0264353 (PMC8880607; doi:10.1371/journal.pone.0264353)
Supplement: S2 Table — (DOCX) [file pone.0264353.s007.docx]

| Variant | After 60 ns of MDs | | | After 80 ns of MDs | | | After 100 ns of MDs | | |
| --- | --- | --- | --- | --- | --- | --- | --- | --- | --- |
|  | **Favored**  **Region** | **Allowed Region** | **Disallowed**  **Region** | **Favored**  **Region** | **Allowed Region** | **Disallowed**  **Region** | **Favored**  **Region** | **Allowed Region** | **Disallowed**  **Region** |
| wtIL-2 | 95.16% | 4.15% | 0.69% | 95.16% | 3.80% | 1.03% | 95.33% | 3.80% | 0.87% |
| M1 | 95.33% | 3.80% | 0.87% | 95.16% | 3.80% | 1.03% | 95.16% | 3.80% | 1.03% |
| M2 | 95.15% | 4.15% | 0.70% | 94.64% | 4.33% | 1.03% | 94.81% | 4.15% | 1.03% |
