# Supplementary material for: Human IL-2Rɑ subunit binding modulation of IL-2 through a decline in electrostatic interactions: A computational and experimental approach
Source: PLoS One. 2022 Feb 25;17(2):e0264353. doi: 10.1371/journal.pone.0264353 (PMC8880607; doi:10.1371/journal.pone.0264353)
Supplement: S3 Table — (DOCX) [file pone.0264353.s008.docx]

| Variant | IL-2Rα | | IL-2Rβ | | IL-2Rγc | |
| --- | --- | --- | --- | --- | --- | --- |
|  | **ΔG (Kcal/mol)** | **Kd (M)** | **ΔG (Kcal/mol)** | **Kd (M)** | **ΔG (Kcal/mol)** | **Kd (M)** |
| wtIL-2 | -11.0 | 9.2E-09 | -10.4 | 2.3E-08 | -10.0 | 4.7E-08 |
| M1 | -10.3 | 2.6E-08 | -10.1 | 3.8E-08 | -10.9 | 1.1E-08 |
| M2 | -9.4 | 1.2E-07 | -11.9 | 1.9E-09 | -8.7 | 4.1E-07 |
