# Supplementary material for: Human IL-2Rɑ subunit binding modulation of IL-2 through a decline in electrostatic interactions: A computational and experimental approach
Source: PLoS One. 2022 Feb 25;17(2):e0264353. doi: 10.1371/journal.pone.0264353 (PMC8880607; doi:10.1371/journal.pone.0264353)
Supplement: Attachment — Submitted filename: Response to the revewers.docx [file pone.0264353.s009.docx]

Dear editor,

Please find enclosed the revised version of our manuscript “Human IL-2Rɑ Subunit Binding Modulation of IL-2 through a Decline in Electrostatic Interactions: A Computational and Experimental Approach” by Arezoo Beig Parikhani et al., which we submit for publication in the PLOSONE.

First, we would like to thank the editor for giving us the opportunity to submit a revised version and the reviewers for their critical but constructive comments on the first version. There have been remarks on the organization of the manuscript and we agree that it could have been done better. Changes to the manuscript are **done with track change** in text. What follows is a point-to-point explanation on how we answered the suggestions and requested amendments:

**Reviewers' comments:**

**Academic editor**

*I have one major concern with the manuscript that I believe can easily be remedy. The results second focuses thoroughly on the computational aspect of the study but failed to mention any of the 'wet biochemistry', that is, the binding studies with M1 and M2. Those aspects were noted in the methods section but not in the results or discussion when the title noted it.*

**Response:** As mentioned in the text of the manuscript, initially insilico studies were performed on M1 and M2 mutant forms and based on the obtained results, M2 mutant was selected for laboratory studies by flow cytometry which represented in experimental section.

*Also, I do not know what M1 and M1 represent: what are the mutations: are they single or multi.*

**Response:** According to the abbreviation list M1 and M2 represent the two mutant forms of human interleukin-2 (Mutant numbers 1 and 2). The triple mutations generated in these two variants have been mentioned in subheading “computational studies” of the result section in which M1 possessed F42A, P65A, and L67A mutations while K35A, F42A, and E61A mutations were selected for M2 molecule. These two variants were further studied and compared with wild type IL-2 molecule.

*There were few typos in the text that I believe will be caught with the revision.*

**Response:** Thanks for your attention. The whole text has been revised.

**Journal Requirements:**

When submitting your revision, we need you to address these additional requirements.

*1- Please ensure that your manuscript meets PLOS ONE's style requirements, including those for file naming.*

**Response:** The manuscript has been fully reviewed according to PLOS ONE's style requirements.

*2- Please note that funding information should not appear in the Acknowledgments section or other areas of your manuscript. We will only publish funding information present in the Funding Statement section of the online submission form. Please remove any funding-related text from the manuscript and let us know how you would like to update your Funding Statement.* *Please include your amended statements within your cover letter; we will change the online submission form on your behalf.*

Response**:** Done.

*3- PLOS ONE now requires that authors provide the original uncropped and unadjusted images underlying all blot or gel results reported in a submission’s figures or Supporting Information files. In your cover letter, please note whether your blot/gel image data are in Supporting Information or posted at a public data repository, provide the repository URL if relevant, and provide specific details as to which raw blot/gel images, if any, are not available. Email us at plosone@plos.org if you have any questions*

**Response:** Done.

*4- Please review your reference list to ensure that it is complete and correct.*

**Response:** The references have been rechecked thoroughly.

**Reviewer #1:**

The authors used computational approach to study IL-2 mutant to potential lower the binding affinity of IL-2 to IL-2R, trying to help design better IL-2 drug to selectively activate effector T cells rather than Treg. The idea is very creative and the study is worth exploring. Just a few questions before it can be published.

*1- IL-2 binds to IL-2Rabg and IL-2Rbg, both provide activation signal to the downstream pathways, when the authors made the mutants. Were the selected amino acids involved in the electrostatic interactions with IL-2Rα also involved in the interactions with IL2Rbg? Would the mutation affect IL2 - IL2Rbg interactions?*

**Response:** Thanks for the question. In order to confirm that decreased affinity is due to the decline in target residual interactions with IL-2Rα, the affinity of wtIL-2 and the mutant forms towards IL-2Rβ and IL-2Rγc were also studied by PRODIGY (S3 table). Although the affinity of M2 to IL-2Rα decreased, an increased affinity was observed towards IL-2Rβ. The assigned mutations affected the affinity of M2 protein to the IL-2Rγc. Since the IL-2Rγc binding site is very close to that of IL-2Rα, mutations in the latter would probably induce conformational changes in the former site. Therefore, docking studies can assume that M2 mutations do not have any significant effect on IL-2 interaction with IL-2Rβ and IL-2γc.

In addition, based on MD studies the alternation in the number of hydrogen bonds between wt-IL2, M1, and M2 with IL-2Rβ, IL-2Rγc, and IL-2Rα were calculated during 100 ns (S2 Fig). In the case of M2 molecule, while the number of hydrogen bonds between M2 and IL-2Rα was the least in comparison with other IL-2 proteins, the interactions were almost constant for IL-2Rβ and IL-2Rγc confirming the neutral effect of the mutations on main interactions between M2 and the two mentioned receptors.

It should also be mentioned that the three IL-2 receptors binding sites are distinct and if the mutations affect M2 interactions with IL-2Rβ and IL-2Rγc (while not significantly according to our studies, it is not directly but inducive.

*2- Since the goal of IL-2 mutein was to avoid Treg expansion, an in vitro experiment would be very helpful to provide further evidence of the mutants that can activate effector T cells only.*

Response: Good comment. Since the aim of the present study was to computationally investigate the effect of triple mutations on binding of IL-2 to the IL-2Rα receptor, the only *in vitro* experiment carried on was flow cytometry analysis. Further experiments are going to perform to study the effects on Tregs in *in vitro* and *in vivo* conditions which will be shown in ongoing manuscript.

*3- A diagram of how the authors performed umbrella sampling would be very helpful.*

Response: Thanks for the suggestion. A diagram of the whole computational study including umbrella sampling has been added to supplementary information file.
